# Machine Learning for Discovery of New ADORA Modulators

**DOI:** 10.3389/fphar.2022.920643

**Published:** 2022-06-22

**Authors:** Ana C. Puhl, Zhan-Guo Gao, Kenneth A. Jacobson, Sean Ekins

**Affiliations:** ^1^ Collaborations Pharmaceuticals, Inc., Raleigh, NC, United States; ^2^ Molecular Recognition Section, Laboratory of Bioorganic Chemistry, National Institute of Diabetes and Digestive and Kidney Diseases, National Institutes of Health, Bethesda, MD, United States

**Keywords:** ADORA, crisaborole, febuxostat, paroxetine, machine learning, adenosine receptor

## Abstract

Adenosine (ADO) is an extracellular signaling molecule generated locally under conditions that produce ischemia, hypoxia, or inflammation. It is involved in modulating a range of physiological functions throughout the brain and periphery through the membrane-bound G protein-coupled receptors, called adenosine receptors (ARs) A_1_AR, A_2A_AR, A_2B_AR, and A_3_AR. These are therefore important targets for neurological, cardiovascular, inflammatory, and autoimmune diseases and are the subject of drug development directed toward the cyclic adenosine monophosphate and other signaling pathways. Initially using public data for A_1_AR agonists we generated and validated a Bayesian machine learning model (Receiver Operator Characteristic of 0.87) that we used to identify molecules for testing. Three selected molecules, crisaborole, febuxostat and paroxetine, showed initial activity *in vitro* using the HEK293 A_1_AR Nomad cell line. However, radioligand binding, β-arrestin assay and calcium influx assay did not confirm this A_1_AR activity. Nevertheless, several other AR activities were identified. Febuxostat and paroxetine both inhibited orthosteric radioligand binding in the µM range for A_2A_AR and A_3_AR. In HEK293 cells expressing the human A_2A_AR, stimulation of cAMP was observed for crisaborole (EC_50_ 2.8 µM) and paroxetine (EC_50_ 14 µM), but not for febuxostat. Crisaborole also increased cAMP accumulation in A_2B_AR-expressing HEK293 cells, but it was weaker than at the A_2A_AR. At the human A_3_AR, paroxetine did not show any agonist activity at 100 µM, although it displayed binding with a K_i_ value of 14.5 µM, suggesting antagonist activity. We have now identified novel modulators of A_2A_AR, A_2B_AR and A_3_AR subtypes that are clinically used for other therapeutic indications, and which are structurally distinct from previously reported tool compounds or drugs.

## Introduction

Adenosine (ADO) is an extracellular signaling molecule generated locally under conditions that produce ischemia, hypoxia, or inflammation and is involved in modulating a range of physiological functions throughout the brain and periphery by activating membrane-bound G protein-coupled receptors (GPCRs) (Borea et al., 2018). There are four subtypes of adenosine receptors (A_1_AR, A_2A_AR, A_2B_AR, and A_3_AR), which are the subject of vigorous drug development directed toward the cyclic adenosine monophosphate (cAMP) and other signaling pathways (Borea et al., 2016; Borea et al., 2018; Jacobson et al., 2019; Effendi et al., 2020). Adenosine receptors (ARs) were first classified according to their differential coupling to adenylate cyclase (AC) to regulate cAMP levels (Borea et al., 2018). Adenosine induces various biological effects associated with each adenosine receptor on the membrane surface of specific cells or tissues (Borea et al., 2018). Based upon sequence similarity and G protein-coupling specificity, A_1_AR and A_3_AR share 49% sequence identity and preferentially couple to Gαi/o to inhibit AC (Effendi et al., 2020), which subsequently inhibits presynaptic glutamate release (Ciruela et al., 2006; Effendi et al., 2020). In contrast, A_2A_AR and A_2B_AR receptors, which are ∼59% identical and couple to Gαs, are able to stimulate AC (Cheng et al., 2017) increasing levels of cAMP (Wardas, 2002). A_1_AR and A_2A_AR receptors possess high affinity for ADO, while A_2B_AR and A_3_AR receptors show relatively lower affinity. A_1_AR has been found to be widely distributed throughout the body. In the brain, it slows metabolic activity by a combination of actions. At neuronal synapses, it reduces synaptic vesicle release. A_1_AR is implicated in sleep promotion by inhibiting wake-promoting cholinergic neurons in the basal forebrain (Elmenhorst et al., 2007). A_1_AR is also present in smooth muscle throughout the vascular system (Fredholm et al., 2001; Tawfik et al., 2005). A_1_AR has antiseizure activity and contributes to neuroprotection in models of neurodegeneration (Effendi et al., 2020). A_1_AR activation under hypoxic conditions leads to inhibition of presynaptic Ca^2+^ influx-related release of transmitters (Wu and Saggau, 1997) such as dopamine, acetylcholine, GABA, and, especially, glutamate, to generate neuroprotection (Stone et al., 2009).

A_2A_AR has been linked to the anti-inflammatory effects of adenosine. Activation of A_2A_AR reduces immune cell migration and produces tissue protection from ischemia/reperfusion injury (Okusa et al., 1999; Ohta and Sitkovsky, 2001). In contrast to other adenosine receptors, A_2B_AR, shows upregulated expression in many pathological conditions, such as inflammation, cancer and hypoxia (Borea et al., 2016; Cekic and Linden, 2016; Gao and Jacobson, 2019). It interacts with Gs to induce the PKA signaling to increase cAMP and can trigger signaling transduction to elevate intracellular Ca^+2^ levels (Effendi et al., 2020). Activation of A_2B_AR in mast cells might be useful in the treatment of asthma (Gao and Jacobson, 2017). A_3_AR is widespread with abundant expression in the lung and liver, and its activation reduces inflammation and chronic neuropathic pain (Jacobson et al., 2019). A_3_AR coupled to Gi proteins inhibits AC, decreases cAMP accumulation and PKA activity, while A_3_AR also increases Ca^2+^ levels and modulates PKC activity (Baraldi et al., 2012; Borea et al., 2018).

ARs are therefore important targets for neurological, cardiovascular, inflammatory, and autoimmune diseases (Borea et al., 2016; Borea et al., 2018). In addition, selective ligands are available for the different AR subtypes, which increase the chances to achieve spatially-specific modulation, representing a pharmacological opportunity to control addictive psychostimulant consumption, among many other health problems (Ballesteros-Yáñez et al., 2017). Initially, we used machine learning models to find agonists of A_1_AR and futher expanded the testing against other subtypes, and we have identified novel AR modulators that are structurally distinct from previously reported tool compounds or drugs in clinical trials for targeting ADO receptors (Jacobson et al., 2019).

## Methods

### Reagents

All test compounds were purchased from MedChemExpress (MCE, Monmouth Junction, NJ).

### Machine Learning

Initially, an A_1_AR model was built with data reported in ChEMBL (Gaulton et al., 2017) (target 262). Assay Central® was used to build the model using EC_50_ values, and non-druglike compounds such as Zn^2+^ (CHEMBL1201279), Li^+^ Cl^−^ (CHEMBL69710), and Li^+^ (CHEMBL1234004) were removed to increase the performance of the model. The ChEMBL compounds for ADORA1 consisted of 430 compounds with EC_50_ values, and the corresponding Bayesian model was built using Assay Central®. Assay Central® has been used by our group in various drug discovery projects (Hernandez et al., 2018; Lane et al., 2018; Russo et al., 2018; Sandoval et al., 2018; Ekins et al., 2019a; Anantpadma et al., 2019; Ekins et al., 2019b; Dalecki et al., 2019; Wang et al., 2019; Ekins et al., 2020); its use as well as clarification on the applicability of the model statistics have been previously described. Metrics such as Receiver Operator Characteristic (ROC), Recall, Precision, F1 Score, Cohen’s Kappa and Matthew’s Correlation Coefficient are generated from internal five-fold cross-validation of the model. To maximize these internal performance statistics, the software can select a reasonable activity threshold, and generate predictions as well as applicability scores for any desired compound. Higher prediction scores are desirable as scores higher than 0.5 are assigned to active compounds (inhibitors). Higher applicability scores are also desirable as they ensure the representation of a given drug in the training set (Clark et al., 2015). The activity threshold for this external dataset was set to 100 nM, and Assay Central™ was used to generate the model performance metrics.

We have also used our updated Assay Central® software which now uses multiple classification algorithms described previously (Lane et al., 2018) as well as multiple regression algorithms including adaboost, Bayesian, elastic net, K-nearest neighbors (knn), random forest, support vector machine and XGboost. We have further curated data available from ChEMBL for not only A_1_AR (ChEMBL226) but also A_2A_AR (ChEMBL251), A_2B_AR (ChEMBL255) and A_3_AR (ChEMB256L). The data on CHEMBL comprise a diverse set of molecules and may comprise both full agonists and positive allosteric modulators (PAMs). For classification models the cut-off was set to 100 nM. 5-fold cross validation was performed for all algorithms except deep learning, which used the removal of 20% of the training set, in a stratified manner, and these were used as external test sets for models trained on the remainder of the data.

### A_1_AR–cAMP Assay

This assay was performed using the screening services of Innoprot (Bizkaia, Spain). For screening of the initial compounds predicted by machine learning models to activate A_1_AR receptor, we used the HEK293 A_1_AR Nomad cell line, which consists of HEK293 cells stably expressing human A_1_AR with no tag. This cell line has been designed to assay compounds or analyze their capability to modulate the A_1_AR. When the agonist binds to A_1_AR in this engineered cell line a G_o_ protein is activated, which in turn, triggers a cellular response mediated by cAMP inhibition. This cellular response can be measured by quantifying the increase in fluorescence intensity and its cellular distribution. An agonist assay was performed for 29 compounds (predicted to be active by the machine learning model) in the human recombinant HEK293 A_1_AR Nomad cell line using a fluorescence-based assay. An agonist effect of the compounds was measured by quantifying the changes in the fluorescence emission cAMP Nomad biosensor, this elevation of fluorescence was analyzed using a plate reader Synergy 2 (Biotek, Winooski, VT). The error bars represent the standard deviation among the three replicate wells. The 29 compounds were tested at 10 μM using nonselective AR agonist adenosine-5′-*N*-ethyluronamide (NECA) at 10 μM as a reference.

### Radioligand Binding Assays

HEK293 cells stably expressing the human A_1_AR, A_2A_AR, A_3_AR were cultured in DMEM supplemented with 10% fetal bovine serum, 100 Units/ml penicillin, 100 µg/ml streptomycin, and 2 µmol/ml glutamine. To prepare cell membranes, cells were detached from culture plates by scraping into cold PBS and centrifuged at 250 g for 5 min. The pellets were resuspended in ice-cold PBS buffer (pH 7.4) and then homogenized. After homogenization and suspension, cells were centrifuged at 1,000 g for 10 min, and the pellet was discarded. The suspension was then re-centrifuged at 20,000 g for 60 min at 4°C. The pellets were resuspended in buffer containing 3 Units/ml adenosine deaminase (Worthington Biochemical, Lakewood, NJ) and incubated at 37°C for 30 min. The aliquots of membrane preparations were stored at −80°C until the binding experiments. For displacement binding assays, membrane preparations (20 µg proteins/tube) were incubated at 25°C for 60 min with a final concentration of [^3^H]DPCPX (0.5 nM), [^3^H]ZM241385 (1.0 nM), [^3^H]DPCPX (5.0 nM) and [^125^I]I-AB-MECA (0.1 nM) for A_1_AR, A_2A_AR, A_3_AR, respectively, in a mixture containing 50 µl of increasing concentrations of a test ligand in a total assay volume of 200 µl of 50 mM Tris HCl, pH 7.4, containing 10 mM MgCl_2_. Nonspecific binding was determined using 100 µM of XAC. The reaction was terminated by filtration with GF/B filters using a Brandel (Gaithersburg, MD) 24-channel harvester. Filters were placed in scintillation vials containing 5 ml of Hydrofluor scintillation buffer (National Diagnostics, Atlanta, GA) and counted using a Tricarb 2810TR liquid scintillation counter (PerkinElmer, Waltham, MA).

### cAMP Assay in AR-Expressing HEK293 Cells

HEK293 cells were grown in 96-well plates in DMEM supplemented with 10% fetal bovine serum, 100 Units/ml penicillin, 100 µg/ml streptomycin, and 2 µmol/ml glutamine. After overnight growth, cells were treated with assay buffer containing phosphodiesterase (PDE) inhibitor rolipram (10 µM), and adenosine deaminase (3 units/ml) for 30 min (for A_1_AR, A_2A_AR and A_3_AR assays PSB603 (8-[4-[4-(4-chlorophenzyl)piperazide-1-sulfonyl)phenyl]]-1-propylxanthine, 1 µM, was included to block the endogenous A_2B_AR) followed by addition of agonists and a 20 min incubation. For A_1_AR and A_3_AR, after incubation with agonists for 20 min, forskolin (10 µM) was added and the mixture incubated for an additional 15 min. The reaction was terminated upon removal of the supernatant and addition of 100 µl Tween-20 (0.3%). Intracellular cAMP levels were measured with an ALPHAScreen cAMP assay kit as instructed by the manufacturer (PerkinElmer).

### A_1_AR–bla U2OS–Agonist Screen

This assay was performed using the screening services of Thermo Fisher (Waltham, MA). A_1_AR-bla U2OS cells are thawed and resuspended in Assay Media (Freestyle media) to a concentration of 312,500 cells/ml. 32 μl of cell suspension (10,000 cells) was added to each well of a 384-well TC-Treated assay plate. Cells in Assay Media were incubated for 16–24 h in the plate at 37°C/5% CO_2_ in a humidified incubator. 4 μl aliquots of a 10X serial dilution of NECA (control agonist starting concentration, 500 nM) or compounds were added to appropriate wells of the plate. 4 μl of Assay Media was added to all wells to bring the final assay volume to 40 μl. The plate was incubated for 5 h at 37°C/5% CO_2_ in a humidified incubator. 8 μl of 1 μM substrate + Solution D Loading Solution was added to each well and the plate was incubated for 2 h at room temperature. The plate was read on a fluorescence plate reader.

### PathHunter^®^ β-Arrestin Assays

This assay was performed using the screening services of Eurofins (Luxembourg). The PathHunter^®^ β-Arrestin assay monitors the activation of a A_1_AR in a homogenous, non-imaging assay format using a technology developed by DiscoverX called Enzyme Fragment Complementation (EFC) with β-galactosidase (β-Gal) as the functional reporter. The enzyme is split into two inactive complementary portions (EA for Enzyme Acceptor and PK for ProLink) expressed as fusion proteins in the cell. EA is fused to β-Arrestin and PK is fused to the A_1_AR. When the A_1_AR is activated and β-Arrestin is recruited to the receptor, ED and EA complementation occurs, restoring β-Gal activity which is measured using chemiluminescent PathHunter^®^ Detection Reagents. PathHunter cell lines were expanded from freezer stocks according to standard procedures. Cells were seeded in a total volume of 20 μl into white walled, 384-well microplates and incubated at 37°C for the appropriate time prior to testing. For agonist determination, cells were incubated with sample to induce response. Intermediate dilution of sample stocks was performed to generate 5X sample in assay buffer. 5 μl of 5X sample was added to cells and incubated at 37°C or room temperature for 90–180 min. Vehicle concentration was 1%. Assay signal was generated through a single addition of 12.5 or 15 μl (50% v/v) of PathHunter Detection reagent cocktail, followed by a 1 h incubation at room temperature. Microplates were read following signal generation with a PerkinElmer Envision™ instrument for chemiluminescent signal detection. Compound activity was analyzed using CBIS data analysis suite (ChemInnovation, San Diego, CA). For agonist mode assays, percentage activity was calculated using the following formula: % Activity = 100% x (mean RLU of test sample–mean RLU of vehicle control)/(mean MAX control ligand–mean RLU of vehicle control).

### A_1_AR–Calcium Influx Assay

This assay was performed using the screening services of Eurofins. Evaluation of the agonist activity of compounds at the human A_1_ receptor expressed in BA/F3 cells was determined by measuring their effect on cytosolic Ca^2+^ ion mobilization using a fluorimetric detection method. The cells were suspended in HBSS buffer (Invitrogen) complemented with 20 mM Hepes and then distributed in microplates at a density of 5 × 10^4^ cells/well. The fluorescent probe (Fluo8, AAT Bioquest, San Francisco, CA) mixed with probenicid in HBSS buffer (Invitrogen) complemented with 20 mM Hepes (Millipore, Burlington, MA) (pH 7.4) was then added into each well and equilibrated with the cells for 60 min at 30°C. Thereafter, the assay plates were positioned in a microplate reader (FlipR Tetra, Molecular Devices, San Jose, CA), which was used for the addition of the test compound, reference agonist or HBSS buffer (basal control), and for the measurements of changes in fluorescence intensity that varies proportionally to the free cytosolic Ca^2+^ ion concentration. For stimulated control measurements, *N* ^6^-cyclopentyladenosine (CPA) at 0.25 μM was added in separate assay wells. The results were expressed as a percent of the control response to CPA at 0.25 μM. The standard reference agonist was CPA, which was tested in each experiment at several concentrations to generate a concentration-response curve from which its EC_50_ value was calculated.

## Results

### Machine Learning

**FIGURE 1:**
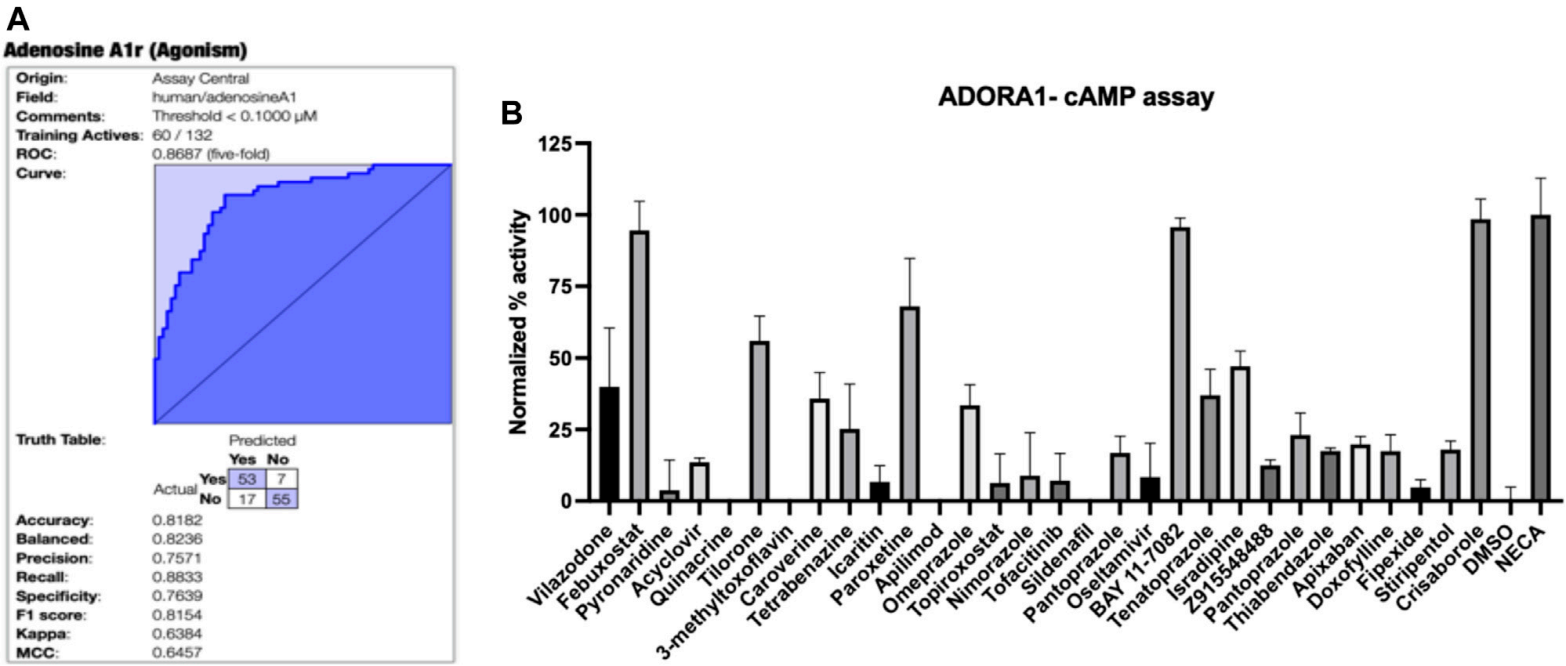
A_1_AR machine learning model and testing of predictions. **(A)** Bayesian machine learning model 5-fold cross validation ROC plot showing statistics for A_1_AR **(B)** Normalized agonist effect of compounds in the activation of A_1_AR receptor. The cells were treated with 30 compounds at 10 µM concentration. Data points represent the mean ± SD for each condition for a single experiment performed in triplicate. The results were normalized to 10 µM NECA and vehicle (DMSO) that were considered to be 100% and 0%, respectively.

Assay Central® (Clark and Ekins, 2015; Clark et al., 2015) is our in-house software that was used to curate the published A_1_AR data, build and validate machine learning models then enable predictions for molecules. This software has been previously used to build Bayesian machine learning models that generate predictions in toxicological and drug discovery projects (Lane et al., 2018; Russo et al., 2018; Zorn et al., 2019). The interpretation of the model metrics as well as the prediction and applicability scores have been described in detailed in previously published work (Clark et al., 2015; Lane et al., 2018; Russo et al., 2018; Zorn et al., 2019). While our Assay Central® software can select a reasonable threshold, 100 nM was set for the original A_1_AR Bayesian model (and subsequent models derived with additional algorithms). Compounds with an EC_50_ lower than this threshold were considered active, and those above were considered inactive. The initial Bayesian model for A_1_AR agonists using literature data (Figure 1A), demonstrated a five-fold cross-validation ROC of 0.87, which is excellent. This model was then used to predict the SuperDrug library (Siramshetty et al., 2018) and our in-house library of compounds (predominantly consisting of FDA approved drugs and other compounds of interest), and 30 compounds were initially selected for testing for agonist activity in the A_1_AR cAMP assay (Supplementary Table S1).

Subsequently we have also built classification and regression models using our latest version of the Assay Central software for A_1_AR, A_2A_AR, A_2B_AR and A_3_AR (Supplementary Figures S1–S4). The classification machine learning models (100 nM cutoff) all had good 5-fold cross-validation statistics, and we have additionally generated regression models that can be used for scoring and selecting new compounds for testing in future. We used these additional models to predict activity of the hits selected from our initial models (Supplementary Table S2).

### A_1_AR Assays

The initially selected 29 molecules were tested using the HEK293 A_1_AR Nomad cell line stably expressing human protein with no tag (Figure 1B). Activation of A_1_AR by an agonist engages G_i1_/_2_/_3_ or G_o_ protein to inhibit adenylate cyclase and, therefore, decreases the cAMP concentration. Compounds were tested at 10 µM using NECA at 10 µM as the reference agonist. The fluorescence intensity was normalized to NECA at 10 µM and vehicle (DMSO) alone, which were considered 100% and 0%, respectively. The normalized results showed that febuxostat, BAY11-7082 ((E)-3-(4-methylphenylsulfonyl)-2-propenenitrile) and crisaborole showed a normalized activity with respect to the positive control of 94.53%, 95.74% and 98.52%, respectively (Figure 1B). Additionally, tilorone hydrochloride and paroxetine hydrochloride showed a normalized activity with respect to the positive control of 55.9 % and 68.0%, respectively (Figure 1B). Other compounds predicted computationally by Assay Central showed an activity of less than 50% at the concentration tested. We therefore conducted additional assays with crisaborole, febuxostat and paroxetine using radioligand binding assays and measured dose response curves in the HEK293 A_1_AR assay using ALPHAScreen (Perkin Elmer) assay, as described below.

### Radioligand Binding

The first step toward activation involves receptor binding. Thus, we have measured the inhibition of the binding of standard AR radioligands at the orthosteric site of three AR subtypes (Table 1). No significant binding inhibition was observed at 100 µM for the three compounds at the human A_1_AR or human A_2B_AR or by crisaborole at human A_2A_AR and A_3_ARs. However, febuxostat and paroxetine both inhibited orthosteric radioligand binding in the µM range for A_2A_AR and A_3_AR.

**TABLE 1:** Inhibition of specific binding at all four ARs (% inhibition at 100 µM of the radioligand shown, or K_i_ (µM)).^a^

Molecule	A_1_ ([^3^H]DPCPX)	A_2A_ ([^3^H]ZM241385)	A_2B_ ([^3^H]DPCPX)	A_3_ ([^125^I]I-AB-MECA)
Crisaborole	36.9 ± 3.4%	31.2 ± 5.6%	<10%	28.8 ± 16.3%
Febuxostat	29.8 ± 5.2%	22.9 ± 4.3 µM	<10%	67.3 ± 45.7 µM
Paroxetine	9.1 ± 3.9%	40.4 ± 9.8 µM	<10%	14.5 ± 9.7 µM

a Data are expressed as mean ± standard error from three independent experiments. Experimental procedures are described in Methods.

### Functional Activity on cAMP Levels in Transfected HEK Cells

**FIGURE 2:**
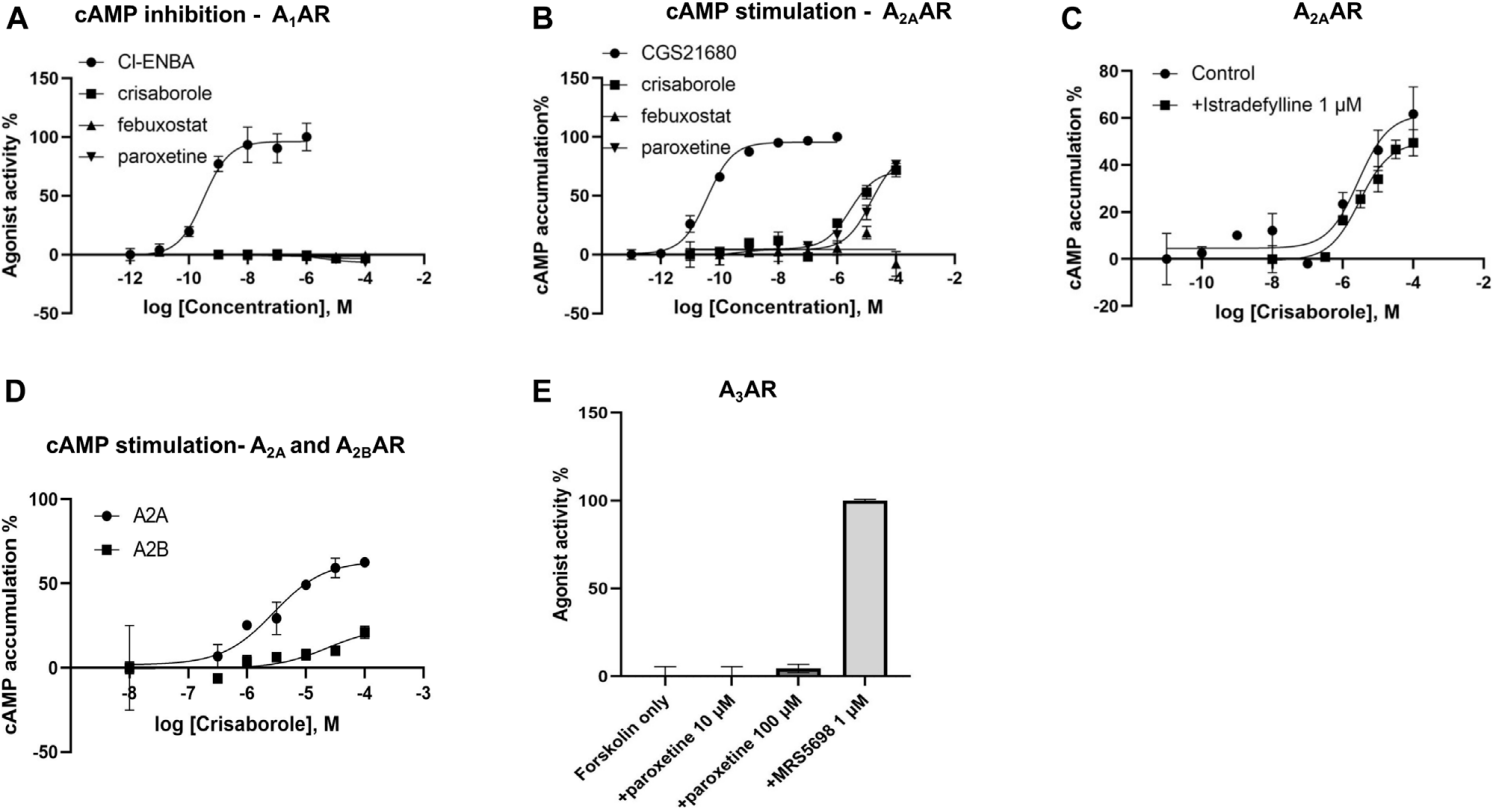
Functional effects measured in transfected HEK cells. **(A)** Determination of cAMP inhibition in A_1_AR-expressing cells, using *N* ^6^-bicyclo [2.2.1]hept-2-yl-5′-chloro-5′-deoxyadenosine (Cl-ENBA) as a reference full agonist (100% stimulation). **(B)** Determination of cAMP stimulation in A_2A_AR-expressing cells, using 2-[p-(2-carboxyethyl)phenyl-ethylamino]-5′-*N*-ethylcarboxamidoadenosine (CGS21680) as a reference full agonist (100% stimulation). **(C)** Lack of antagonism, by an orthosteric agonist istradefylline, of the crisaborole-induced cAMP stimulation in A_2A_AR-expressing cells. A_2A_ activation by crisaborole in the absence of antagonist showed an EC_50_ = 2.78 µM, and in the presence of istradefylline EC_50_ = 2.99 µM. **(D)** Comparison of cAMP stimulation by crisaborole in A_2A_- and A_2B_AR-expressing cells, using NECA as a reference full agonist (100% stimulation). **(E)** Lack of agonist effect of paroxetine in A_3_AR-expressing cells, in comparison to (1S,2R,3S,4R,5S)-4-(6-((3-chlorobenzyl)amino)-2-((3,4-difluorophenyl)ethynyl)-9H-purin-9-yl)-2,3-dihydroxy-N-methylbicyclo [3.1.0]hexane-1-carboxamide (MRS5698) as a reference full agonist (100% stimulation).

Functional activity on cAMP levels in transfected HEK cells was determined for the three hit compounds. No A_1_AR agonist activity was observed (Figure 2). In HEK cells expressing the human A_2A_AR, stimulation of cAMP was observed for crisaborole (EC_50_ 2.8 µM) and paroxetine (EC_50_ 14 µM), but not for febuxostat. We did not measure A_2B_AR and A_3_AR effects of febuxostat in the cAMP cell assays. However, crisaborole is not an orthosteric A_2A_AR agonist, because it did not inhibit binding. Istradefylline, an A_2A_AR antagonist, only minimally affected crisaborole’s effect, suggesting a mechanism of crisaborole-induced cAMP accumulation that is different from standard full agonist NECA. Istradefylline (1 µM) had minimum effect, but it slightly lowered both basal value and the maximum effect. This concentration of istradefylline (K_i_ = 2.2 nM) should be sufficient to saturate the orthosteric A_2A_AR binding site. Curiously, crisaborole also increased cAMP accumulation in A_2B_AR-expressing HEK293 cells, but it was weaker than at the A_2A_AR. At the human A_3_AR, paroxetine did not show any agonist activity at 100 µM, although it displayed a binding K_i_ value of 14.5 µM, suggesting antagonist activity. A summary table of *in vitro* data for compounds tested against adenosine receptors is showed in Table 2.

**TABLE 2:** Summary table of *in vitro* data for compounds tested against adenosine receptors.

Adenosine receptor	Binds with	Adenosine	Adenylate cyclase/cAMP	Crisaborole cAMP	Febuxostat cAMP	Paroxetine cAMP
A_1_AR	Gi	High affinity	Inhibition/Decrease	Not active	Not active	Not active
A_2A_AR	Gs	High affinity	Stimulate/Increase	Active E_max_ = 67%	Not active	Active E_max_ = 69%
A_2B_AR	Gs	Low affinity	Stimulate/Increase	E_max_ = 17%	N/A	N/A
A_3_AR	Gi	High affinity	Inhibition/Decrease	N/A	N/A	Not active

### A_1_AR–β-Arrestin Assay

We used A_1_AR-*bla* U2OS cells to test activation of A_1_AR receptor by crisaborole and paroxetine. This parental cell line stably expresses a beta‐arrestin/TEV protease fusion protein and the beta‐lactamase reporter gene under the control of a UAS response element. Paroxetine and crisaborole showed no activation in this system (Supplementary Figure S5). We also used a secondary assay PathHunter^®^ β-Arrestin assay, which monitors the activation of A_1_AR in a homogenous, non-imaging assay format using a technology developed by DiscoverX called Enzyme Fragment Complementation (EFC) with β-galactosidase (β-Gal) as the functional reporter. This data was normalized to the maximal and minimal response observed in the presence of control agonist CPA and vehicle (Supplementary Figure S6), and no activation of the A_1_AR receptor was observed in this assay at the maximum crisaborole concentration of 20 µM.

### A_1_AR–Calcium Influx Assay

Crisaborole was tested using cellular and nuclear receptor functional Assays (Eurofins) for calcium influx assay and showed no activity of the A_1_AR receptor at the maximum concentration tested (20 μM) (Supplementary Figure S7).

## Discussion

While there have been several previous attempts to use machine learning for ARs (Saad et al., 2019; Wang et al., 2021), few have performed external validation. One recent study used deep learning combined with pharmacophore and docking approaches to identify novel A_1_/A_2A_ antagonists (Wang et al., 2021). In contrast, we were keen to use machine learning alone to potentially repurpose existing drugs for ARs. Using our initial machine learning model, we have identified crisaborole as weakly binding to A_1_AR, but without activity on the cAMP, β-arrestin and calcium influx assays. However, crisaborole can activate A_2A_AR and A_2B_AR at the highest concentrations examined. Unexpectedly, the presence of an orthosteric A_2A_AR antagonist istradefylline did not antagonize the effect of crisaborole, suggesting a mechanism of crisaborole-induced cAMP accumulation that is different from standard full agonist NECA. Paroxetine induced weak activation of A_2A_AR, but no activation of A_3_AR, despite a binding K_d_ of 14.5 ± 9.7 µM. The lack of A_3_AR activation suggested that paroxetine is an antagonist at this subtype. The structures of the three hit compounds do not resemble AR-targeting drugs that have been studied in clinical trials previously (Jacobson et al., 2019) (Supplementary Figure S8).

We evaluated the activity of crisaborole using several different *in vitro* assays. Crisaborole is an inhibitor of phosphodiesterase 4 (PDE4), which is responsible for the hydrolysis and subsequent inactivation of cyclic nucleotides such as cAMP. A_1_AR activation promotes inhibition of adenylate cyclase and consequently inhibits cAMP production leading to the inhibition of presynaptic glutamate release (Wardas, 2002). Thus, since crisaborole is an inhibitor of PDE4 and A_1_AR, it may have different effects on cAMP levels that are antagonistic. When crisaborole was tested in a second independent β-arrestin assay using the A_1_AR-*bla* U2OS Cells and PathHunter technology, it showed no agonist activity at the A_1_AR (Supplementary Figures S5, S6) and had no activity in the calcium influx assay (Supplementary Figure S7).

In the United States, crisaborole is indicated for topical treatment of mild to moderate atopic dermatitis in people 3 months of age and older (Schlessinger et al., 2020). Crisaborole enhances cellular control of inflammation by inhibiting PDE4 and its ability to degrade intracellular cAMP. Apparent A_2A_AR agonist-like activity of crisaborole in combination with its PDE4 inhibitory activity may contribute when used topically in the clinic, regardless of the mechanism of A_2A_AR activation.

The medicinal chemistry surrounding the development of novel adenosine receptor ligands has largely been driven by derivatization of the adenosine and other purine-like scaffolds to gain understanding of the structure-activity relationships especially in the early stages to distinguish between A_1_AR and A_2A_AR (Geldenhuys et al., 2017). From these studies, novel scaffolds were developed, such as the A_2A_AR antagonist core 8-styrylxanthine. It was discovered that substitution of the styryl moiety with an 8-phenoxymethyl moiety leads to a dual A_1_/A_2A_ receptor antagonist (Harmse et al., 2016). The current study provides additional scaffolds based on approved drugs that could be modified in the future to improve activities against these receptors.

The A_2A_AR receptor is also expressed in the brain, where it has important roles in the regulation of glutamate and dopamine release, making it a potential therapeutic target for the treatment of conditions such as insomnia, pain, depression, and Parkinson’s disease (Borea et al., 2018). A_2A_AR is recognized as the main adenosine subtype located in the striatum, where it is colocalized with dopamine D_2_ receptors (D_2_R). This results in A_2A_AR/D_2_R heteromers that have a crucial role in the modulation of motor function (Borea et al., 2018). A_2A_AR may be a therapeutic target in Alzheimer’s disease, Huntington’s disease, epilepsy, acute and chronic stress, and memory fear (Borea et al., 2018). Pharmacological agents that increase the activation of A_1_AR in response to adenosine would be useful for the treatment of CNS, cardiovascular, and inflammatory pathologies (Borea et al., 2018). Coactivation of two AR subtypes might be therapeutically beneficial, such as both A_1_AR and A_3_AR in cardioprotection (Jacobson et al., 2005). Understanding the mechanisms of drug actions at GPCRs and translating this understanding into more selective and effective medicines remains a challenge (May et al., 2007). The effects of an allosteric modulator on ligand efficacy and on affinity at the orthosteric site do not always correlate, such that a modulator can increase the affinity of an orthosteric ligand while decreasing the efficacy and vice versa (May et al., 2007).

## Conclusion

The goal of this work was to use machine learning approaches to assist in identifying new molecules to modulate ARs. In the process we have identified several approved drugs with *in vitro* functional activity against the A_2A_AR, A_2B_AR and A_3_AR subtypes, which could potentially be repurposed. The three molecules derived from machine learning each had a distinct pharmacological activity, which diverged in the different *in vitro* assays used and, in some cases, suggested non-canonical interaction with these receptors. Subsequent pharmacological characterization, including the use of AR-knockout mice (Xiao et al., 2019), will be needed to better understand their respective modes of action in future. The data generated in this study may also be used to improve our machine learning models and provide further structural diversity for starting points for medicinal chemistry efforts for AR modulators.

## Data Availability

The original contributions presented in the study are included in the article/Supplementary Material, further inquiries can be directed to the corresponding authors.

## Abbreviations

ADO, Adenosine; ARs, Adenosine receptors; A_1_AR, Adenosine receptor A1; A_2A_AR, Adenosine receptor A2A; A_2B_AR, Adenosine receptor A2B; A_3_AR, Adenosine receptor A3; GPCR, G protein-coupled receptor; cAMP, cyclic adenosine monophosphate; AC, adenylate cyclase; NECA, adenosine-5′-N-ethyluronamide; CPA, N*6*-cyclopentyladenosine; PDE4, phosphodiesterase 4; D_2_R, dopamine D2 receptors.
