# Supplementary material for: Machine Learning for Discovery of New ADORA Modulators
Source: Front Pharmacol. 2022 Jun 22;13:920643. doi: 10.3389/fphar.2022.920643 (PMC9257522; doi:10.3389/fphar.2022.920643)
Supplement: Supplementary file 1 [file DataSheet1.DOCX]

**Supplemental data**

**Machine Learning for Discovery of New ADORA Modulators**

Ana C. Puhl^1^, Zhan-Guo Gao^2^, Kenneth A. Jacobson^2^ and Sean Ekins^1^

^1^Collaborations Pharmaceuticals, Inc., 840 Main Campus Drive, Lab 3510, Raleigh, NC 27606, USA.

^2^Molecular Recognition Section, Laboratory of Bioorganic Chemistry, National Institute of Diabetes and Digestive and Kidney Diseases, National Institutes of Health, Bethesda, MD, 20892, USA

*To whom correspondence should be addressed. Email: [ana@collaborationspharma.com](mailto:ana@collaborationspharma.com)

[sean@collaborationspharma.com](mailto:sean@collaborationspharma.com) Phone: 215-687-1320.

**Short running tile:** New ADORA modulators

**Table S1.** Compounds selected for testing against A_1_AR using machine learning predictions.

| **Structure** | **Name** | **Prediction score** | **Model applicability** | **Average**  **Regression models**  **(-log M)** |
| --- | --- | --- | --- | --- |
| **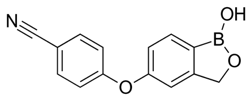** | Crisaborole | 0.95 | 0.29 | 6.96 |
| **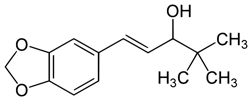** | Stiripentol | 0.91 | 0.50 | 5.66 |
| **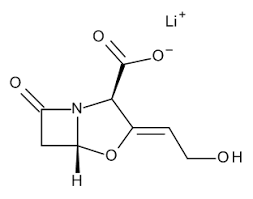** | Fipexide Hydrochloride | 1.04 | 0.65 | 6.22 |
| **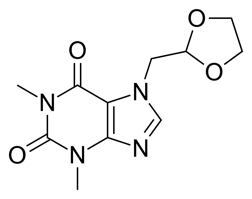** | Doxofylline | 0.85 | 0.40 | 6.51 |
| **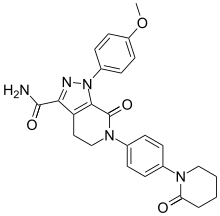** | Apixaban | 0.87 | 0.36 | 6.20 |
| **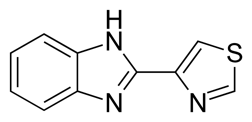** | Thiabendazole | 0.85 | 0.39 | 5.90 |
| **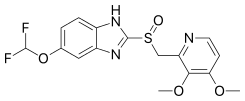** | Pantoprazole | 0.84 | 0.27 | 6.04 |
| **** | Z915548488 | 0.62 | 0.49 | 6.33 |
| **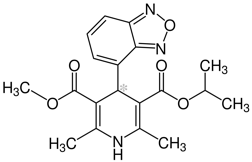** | Isradipine | 0.62 | 0.28 | 5.89 |
| **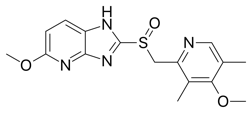** | Tenatoprazole | 0.82 | 0.23 | 6.22 |
| **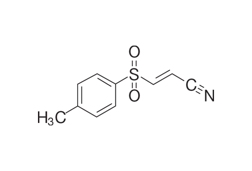** | BAY 11-7082 | 0.80 | 0.36 | 7.05 |
| **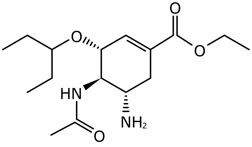** | Oseltamivir Phosphate | 0.65 | 0.42 | 5.86 |
| **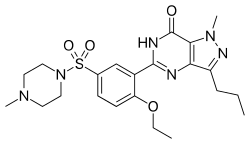** | Sildenafil | 0.73 | 0.30 | 6.93 |
| **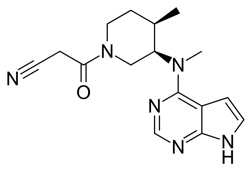** | Tofacitinib Citrate | 0.85 | 0.32 | 7.36 |
| **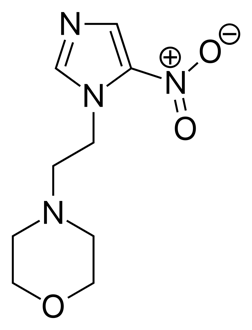** | Nimorazole | 0.90 | 0.43 | 6.04 |
| **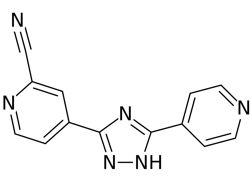** | Topiroxostat | 0.92 | 0.29 | 7.27 |
| **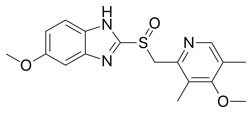** | Omeprazole | 0.88 | 0.28 | 6.29 |
| **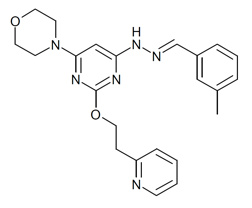** | Apilimod | 0.86 | 0.42 | 6.62 |
| **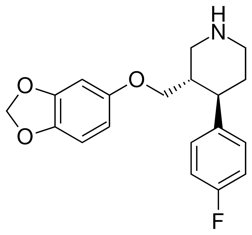** | Paroxetine Hydrochloride | 1.02 | 0.44 | 5.87 |
| **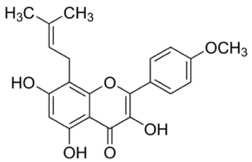** | Icaritin | 0.96 | 0.39 | 6.03 |
| **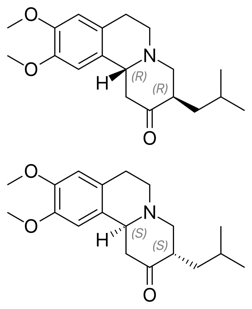** | Tetrabenazine | 0.80 | 0.32 | 5.89 |
| **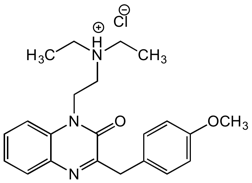** | Caroverine Hydrochloride Monohydrate | 0.86 | 0.49 | 6.00 |
| **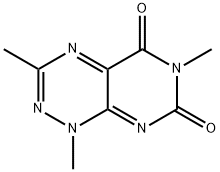** | 3-Methyl Toxoflavin | 0.80 | 0.27 |  |
| **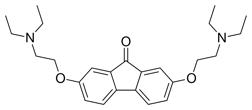** | Tilorone Hydrochloride | 0.87 | 0.52 | 6.37 |
| **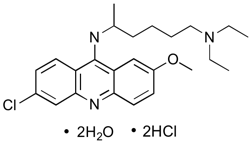** | Quinacrine Dihydrochloride Dihydrate | 0.78 | 0.44 | 6.20 |
| **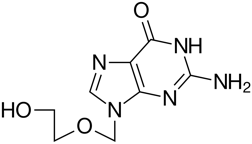** | Acyclovir | 0.88 | 0.41 | 6.12 |
| **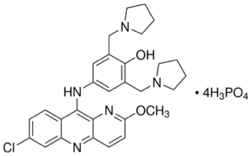** | Pyronaridine Tetraphosphate | 0.77 | 0.38 | 6.20 |
| **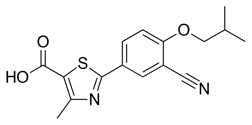** | Febuxostat | 0.78 | 0.46 | 6.53 |
| **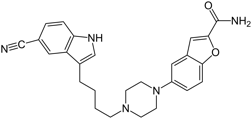** | Vilazodone | 0.77 | 0.35 | 7.39 |

**Table S2:** Classification and regression machine learning model predictions for crisaborole, febuxostat and paroxetine. Prediction values for regression models are shown as -logM and classification models are shown in parenthesis, 0 represents inactive and 1 active at a cut off 100 nM.

| **Molecule** | **A_1_AR** | **A_2A_AR** | **A_2B_AR** | **A_3_AR** |
| --- | --- | --- | --- | --- |
| Crisaborole | 6.96 (0) | 6.13 (0) | 6.49 (1) | 6.75 (0) |
| Febuxostat | 6.53 (0) | 6.26 (0) | 6.82 (0) | 7.24 (0) |
| Paroxetine | 6.05 (0) | 5.73 (0) | 6.54 (1) | 7.03 (0) |

**Figure S1.** Additional regression and classification machine learning models built for A_1_AR using the latest Assay Central software.

**A_1_AR – regression models**

ADORA1 regression models were built with data from ChEMBL (CHEMBL226). The dataset contained 287 molecules, and values ranging from -logM 4.00-10.4. All models present a good R^2^.


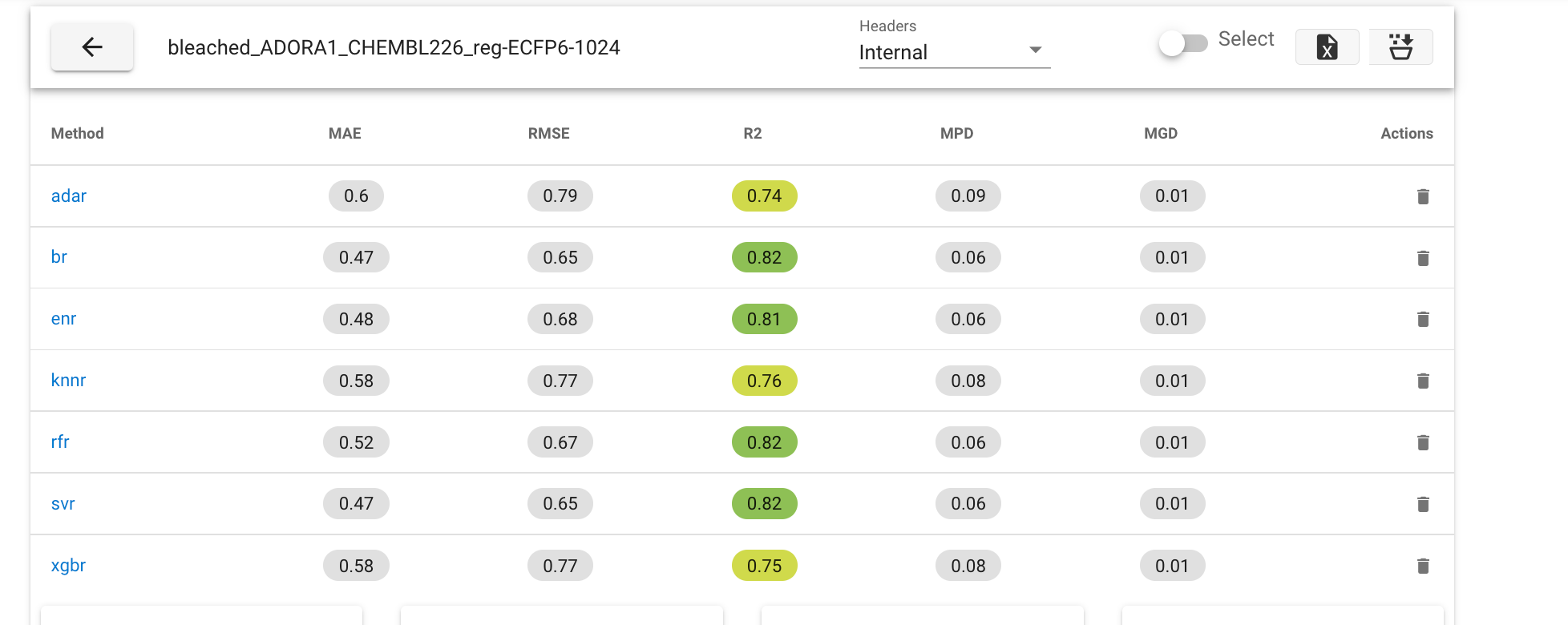


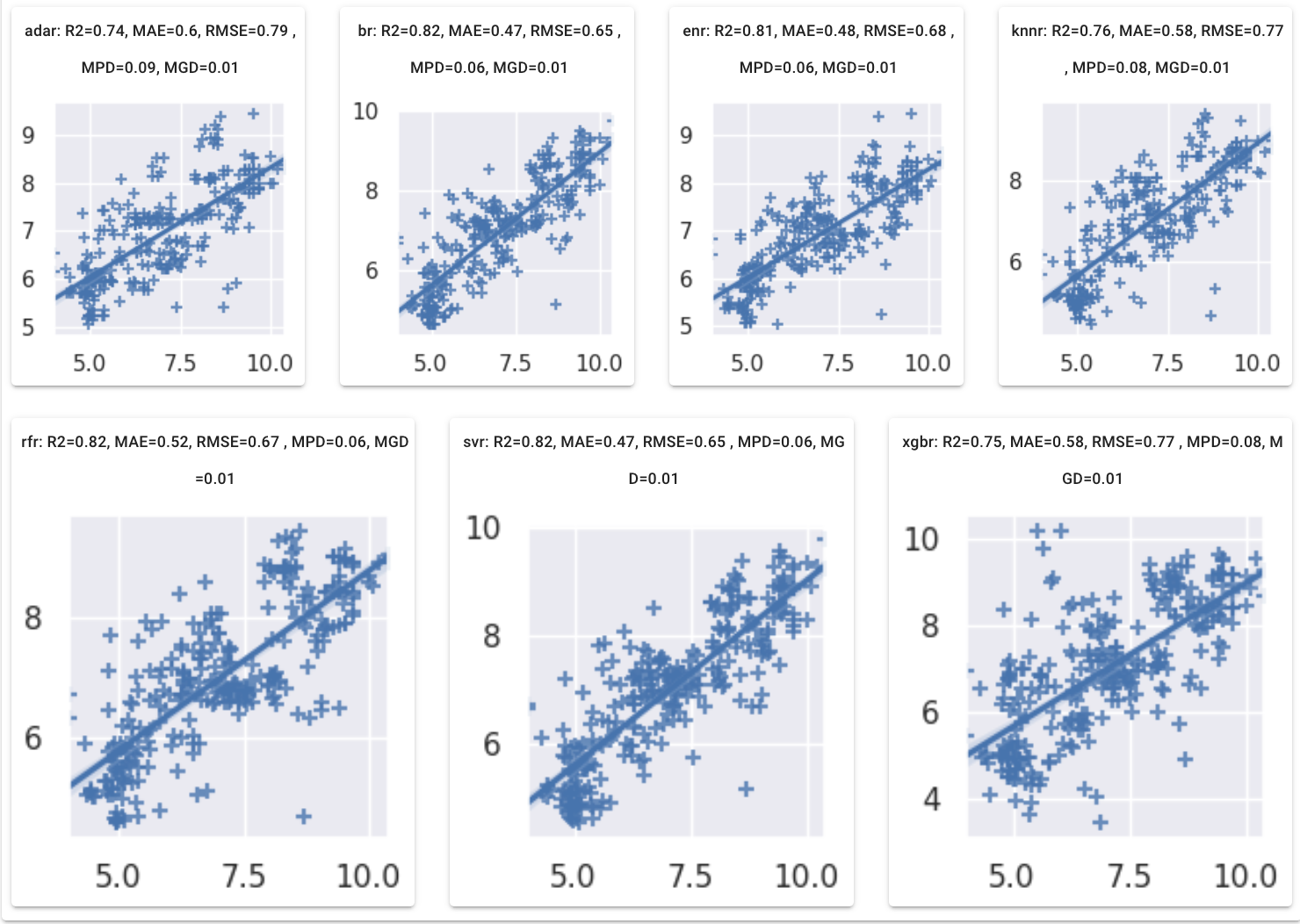


**A_1_AR- classification models**

For classification models, the dataset of 329 molecules was created with a threshold of 100 nM, which resulted in 141 active and 188 inactive molecules, that were used to build the models.


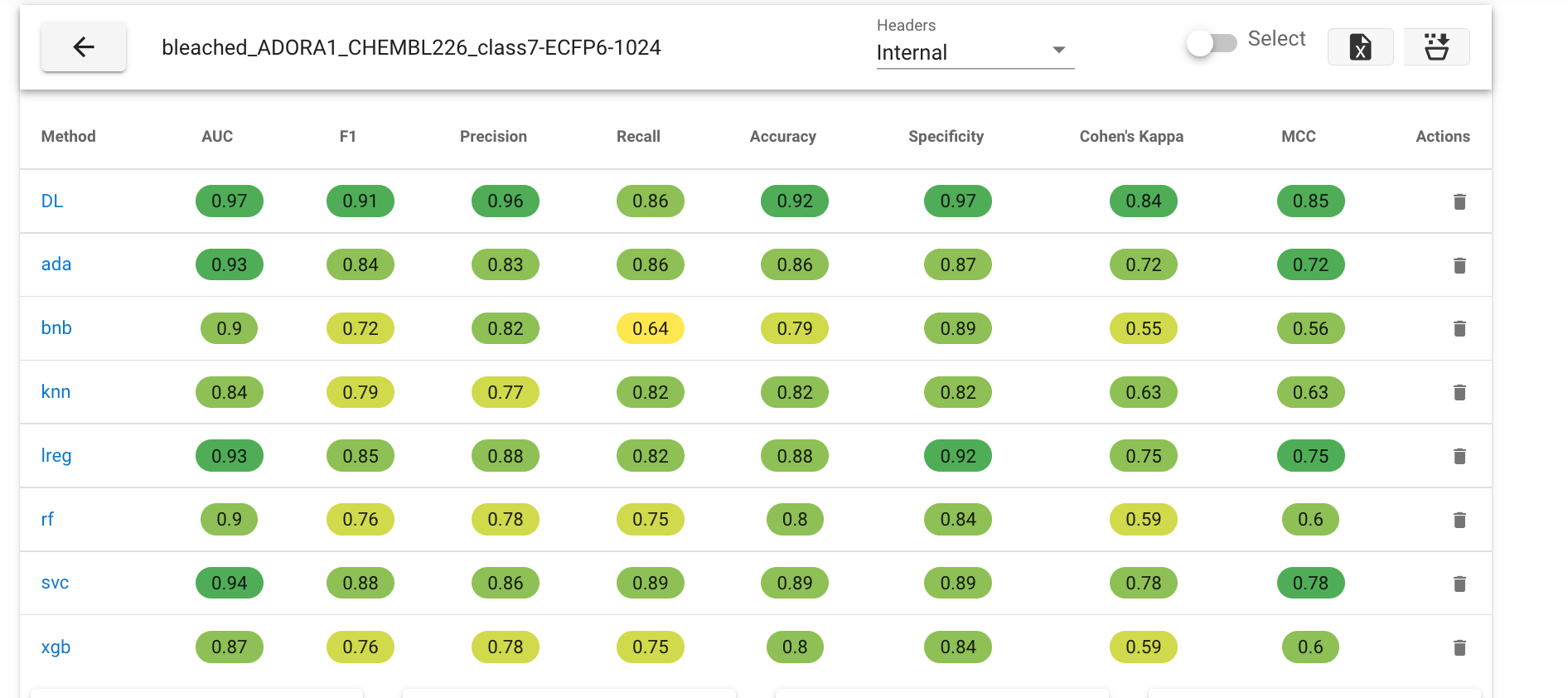


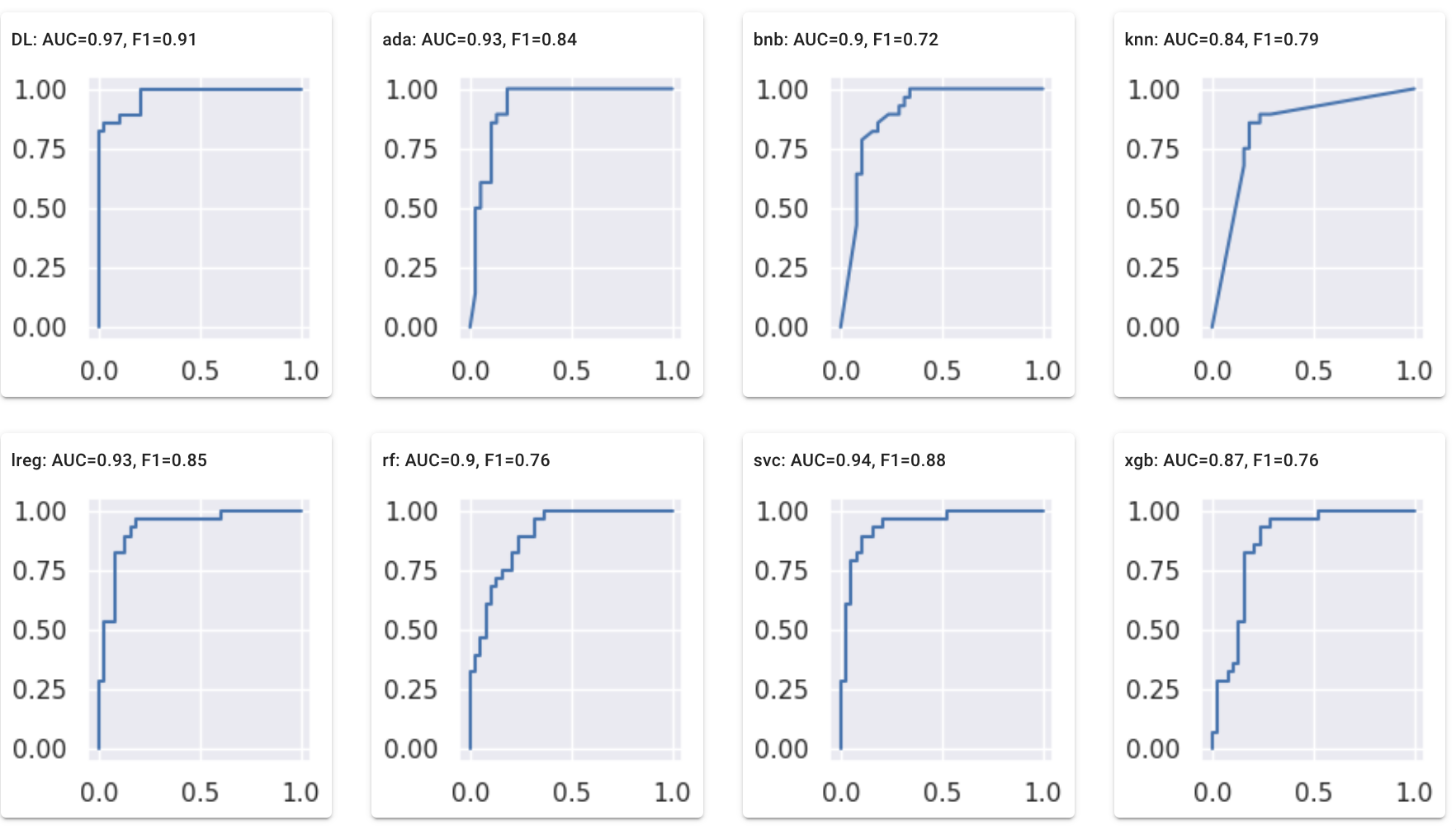


**Figure S2.** Regression and classification machine learning models built for A_2A_AR using the latest Assay Central software.

**A_2A_AR – regression models**

A_2A_AR regression models were built with data from ChEMBL (CHEMBL251). The dataset contained 191 molecules, and values ranging from -logM 4.02-9.19. RFR appears to have the best R^2^.


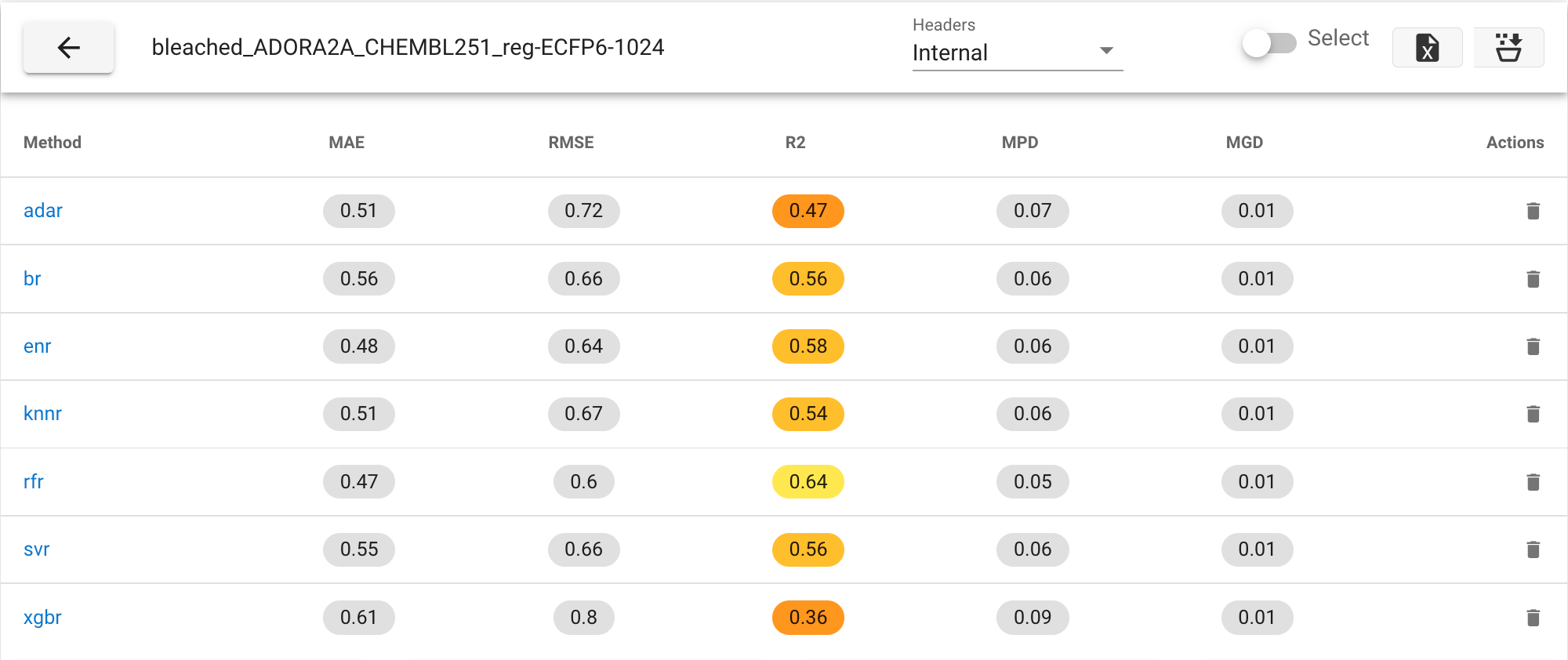


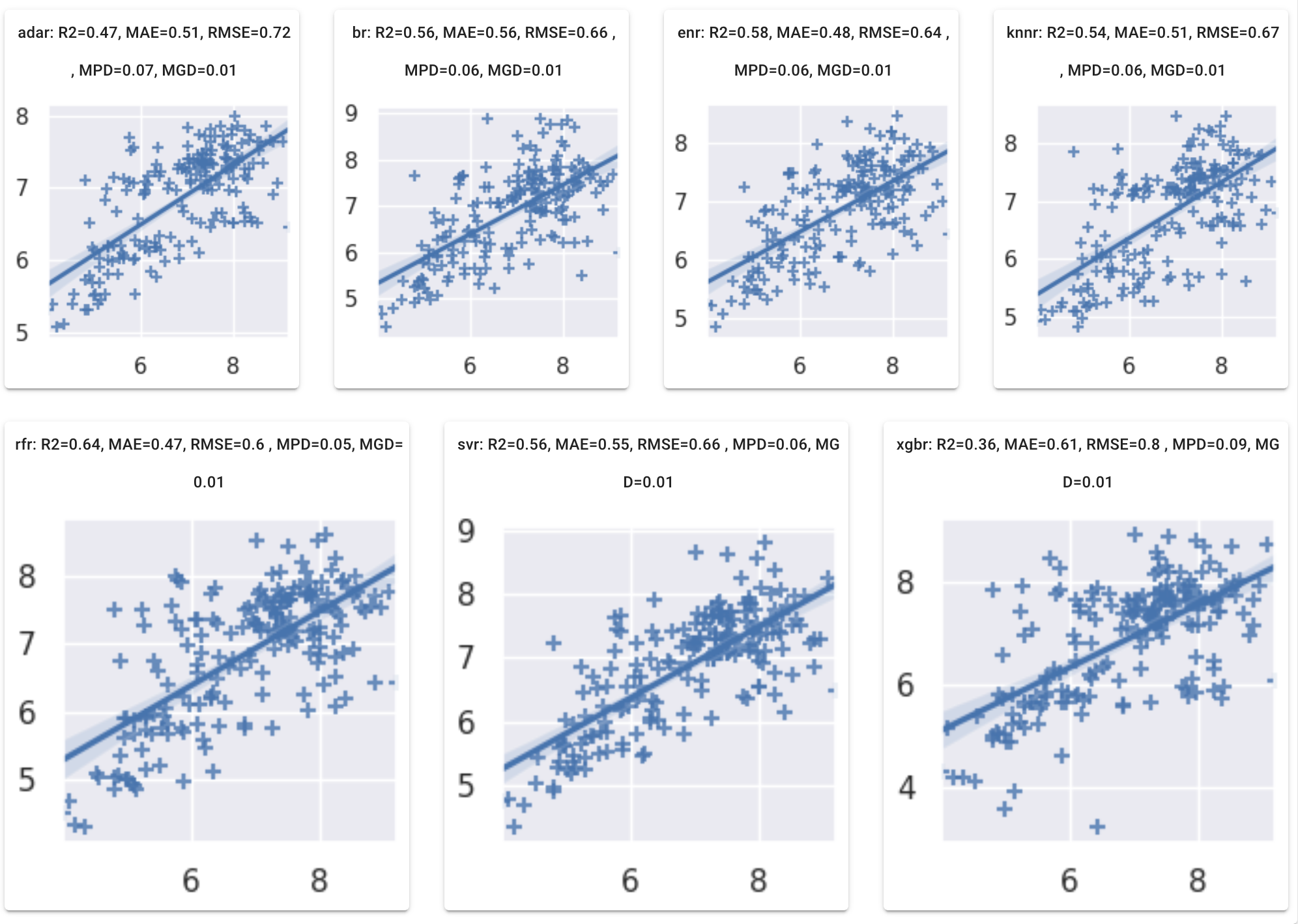


**A_2A_AR – classification models**

For classification models, the dataset of 261 molecules was created with a threshold of 100 nM, which resulted in 104 active and 157 inactive molecules, that were used to build the models.


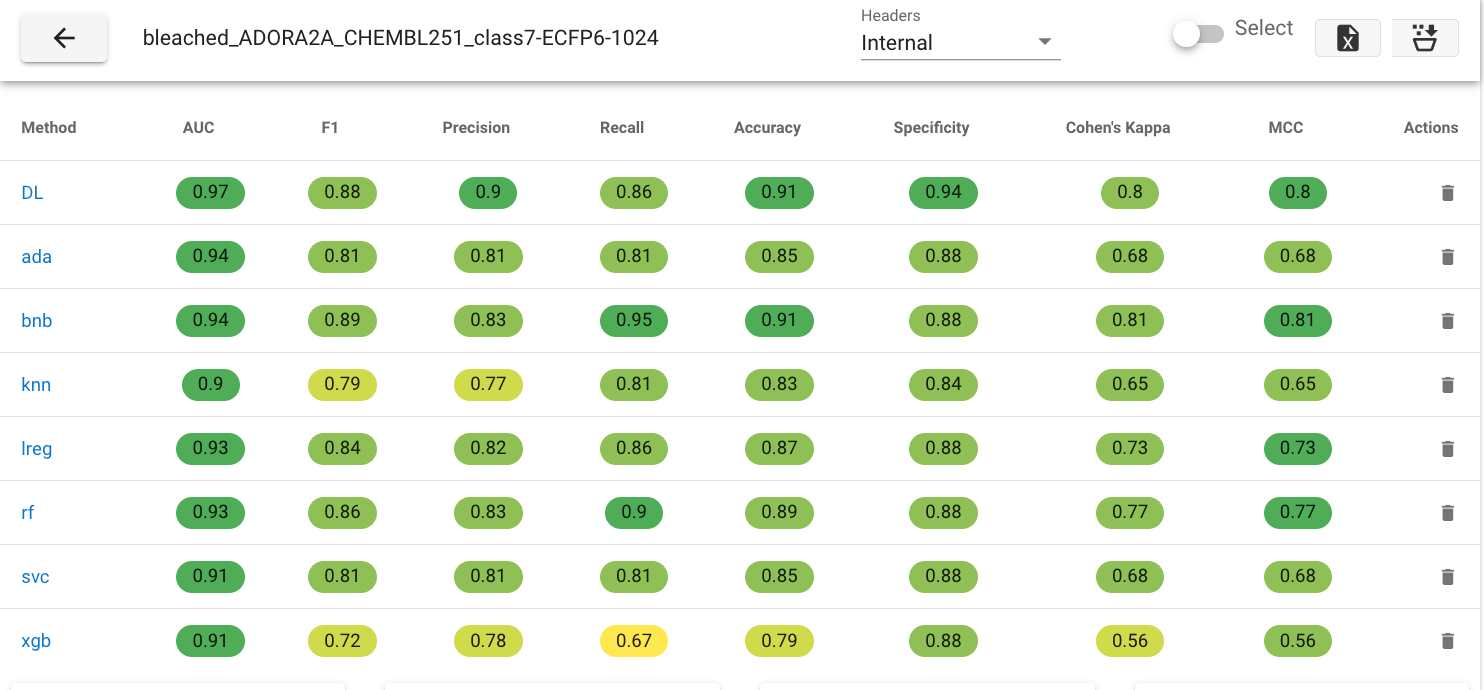


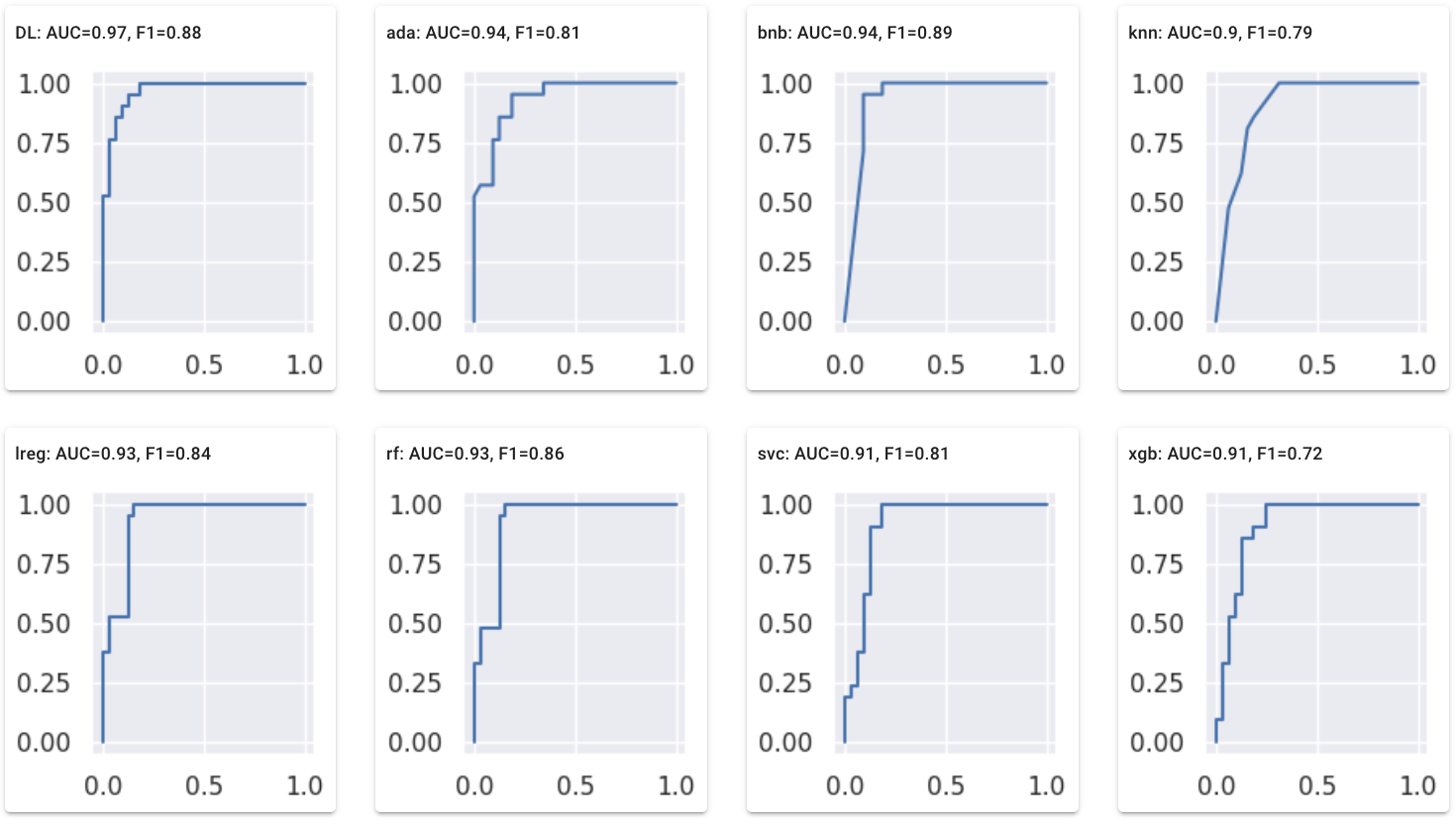


**Figure S3.** Regression and classification machine learning models built for A_2B_AR using the latest Assay Central software.

**A_2B_AR – regression models**

A_2B_AR regression models were built with data from ChEMBL(CHEMBL255). The dataset contained 191 molecules, and values ranging from -logM 4.00-8.64. SVR appears to have the best R^2^.

**
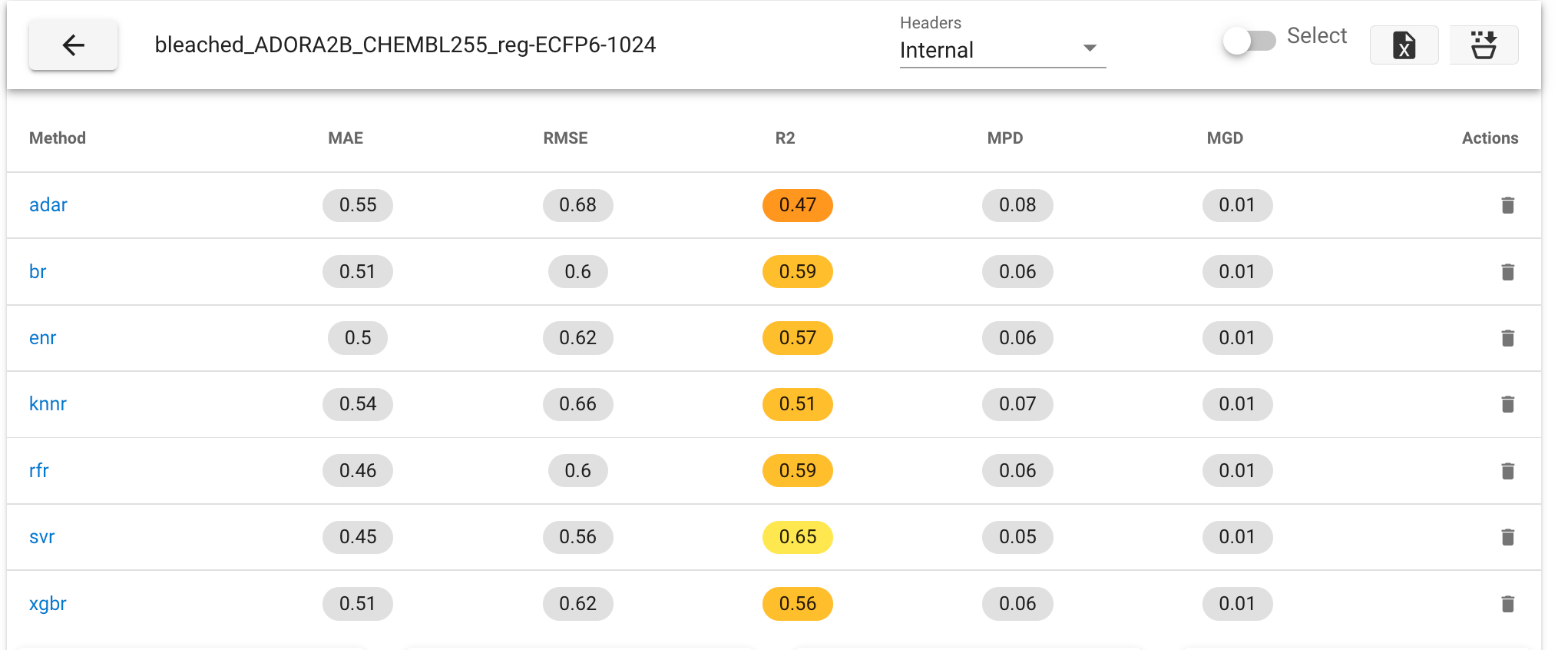
**

**
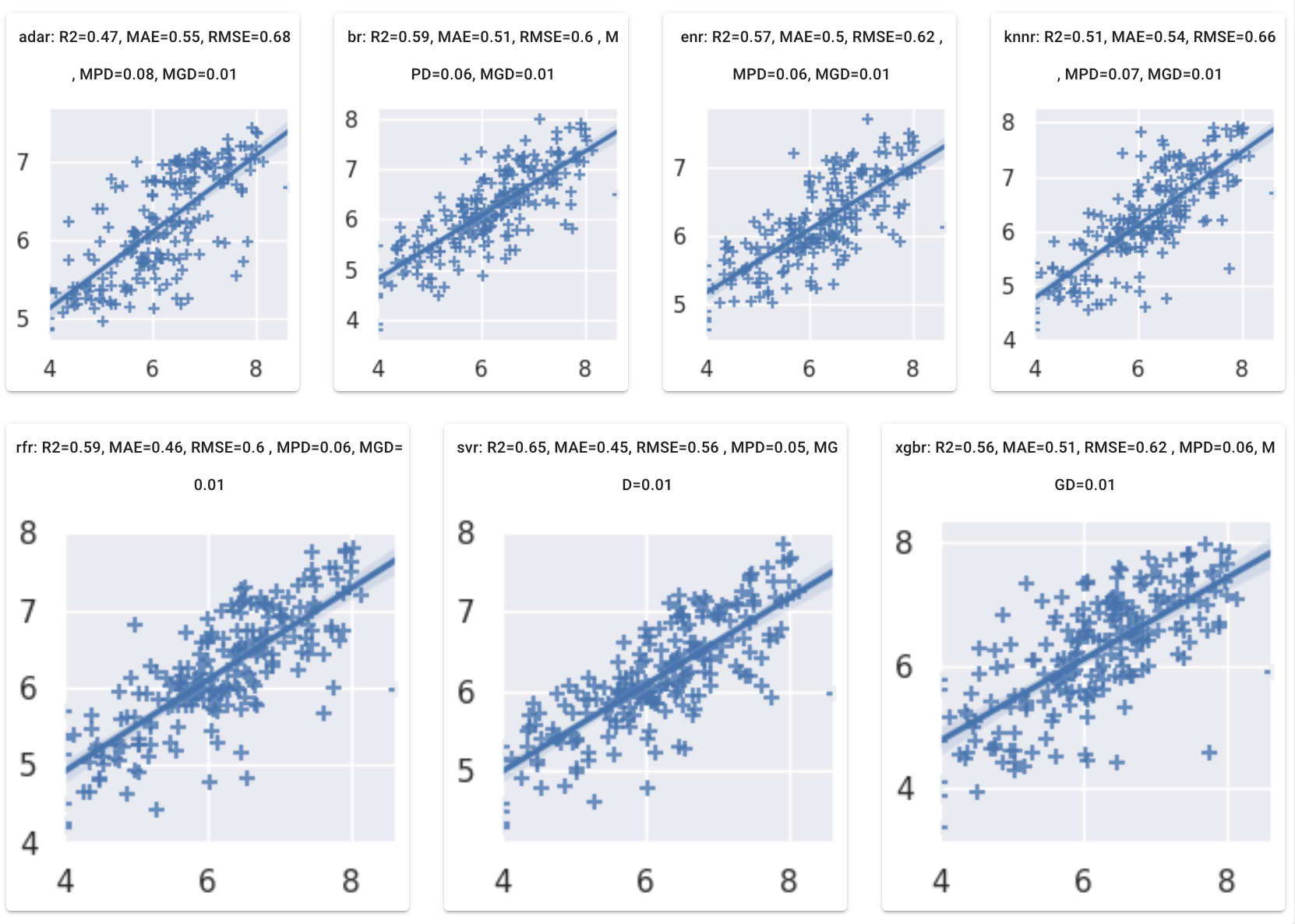
**

**A_2B_AR – classification models**

For classification models, the dataset of 340 molecules was created with a threshold of 100 nM, which resulted in 44 active and 296 inactive molecules, that were used to build the models.

**
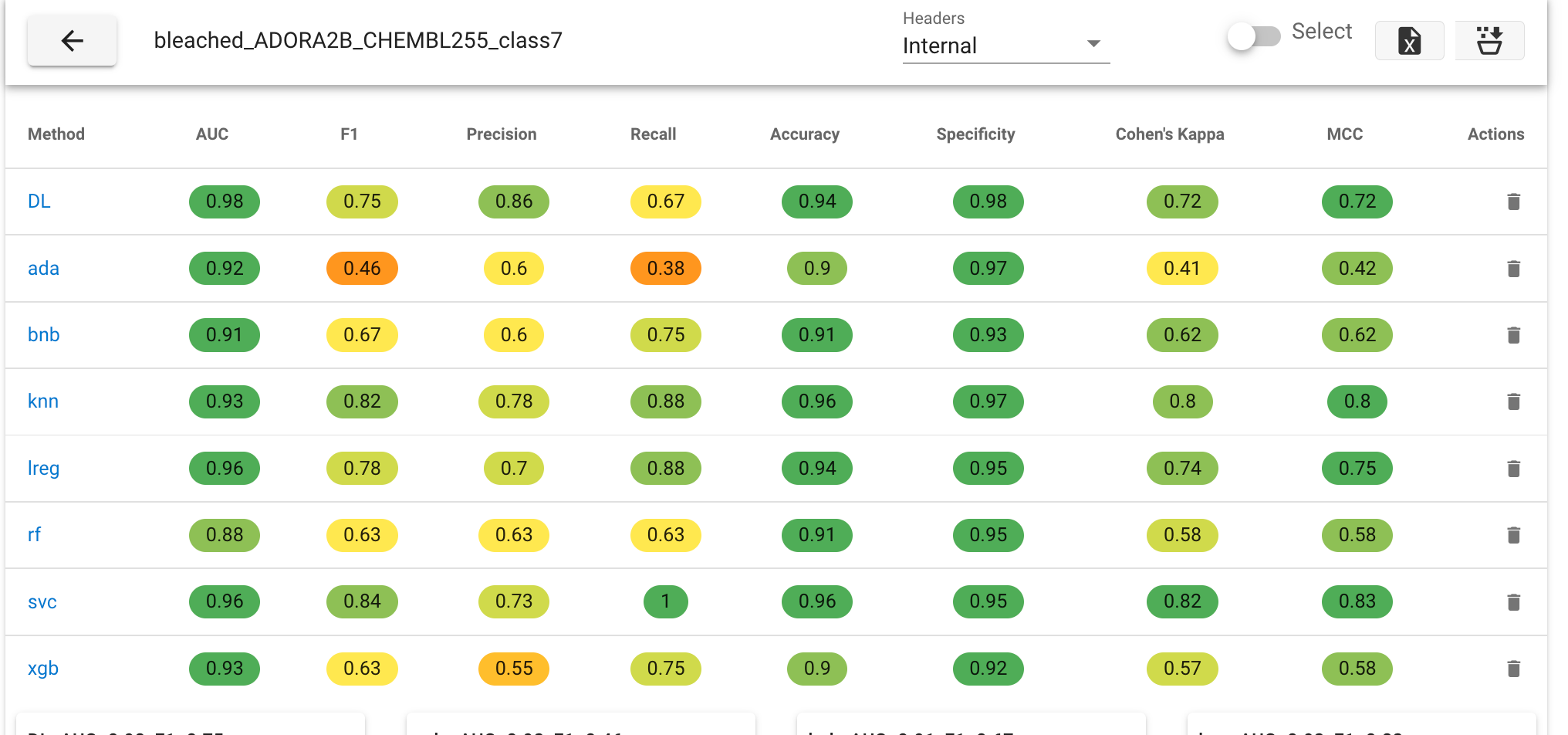
**

**
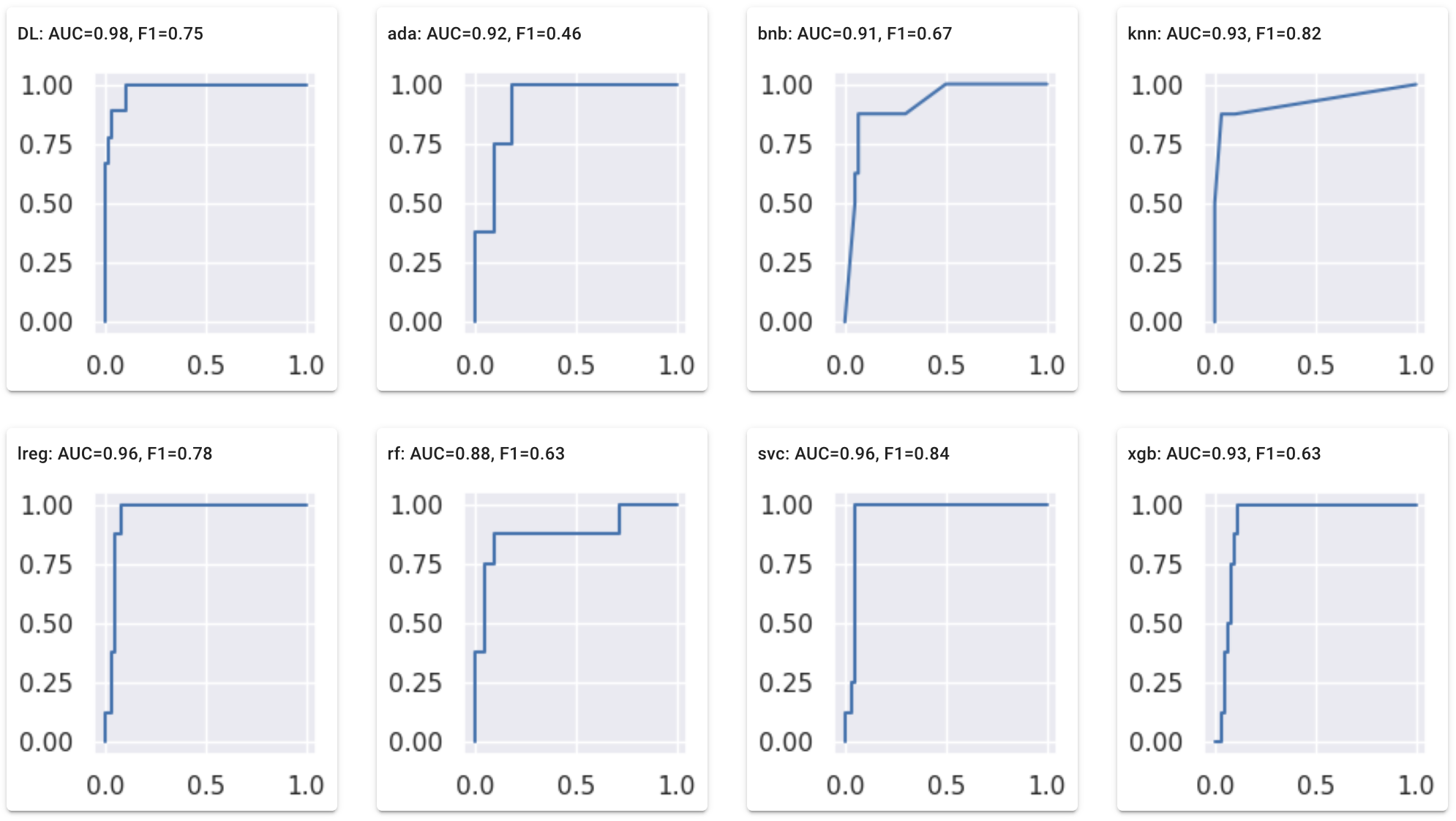
**

**Figure S4.** Regression and classification machine learning models built for A_3_AR using the latest Assay Central software.

**A_3_AR – regression models**

A_3_AR regression models were built with data from ChEMBL (CHEMBL256). The dataset contained 142 molecules, and values ranging from -logM 4.59-9.87.


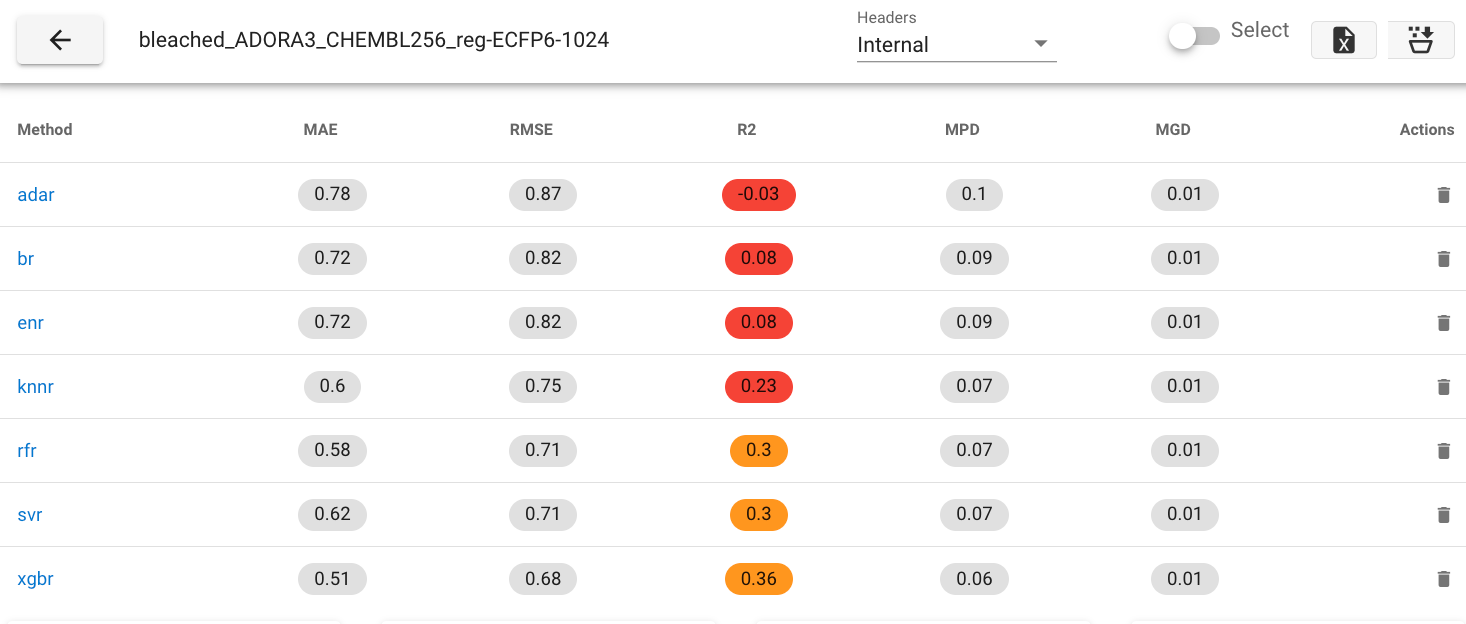


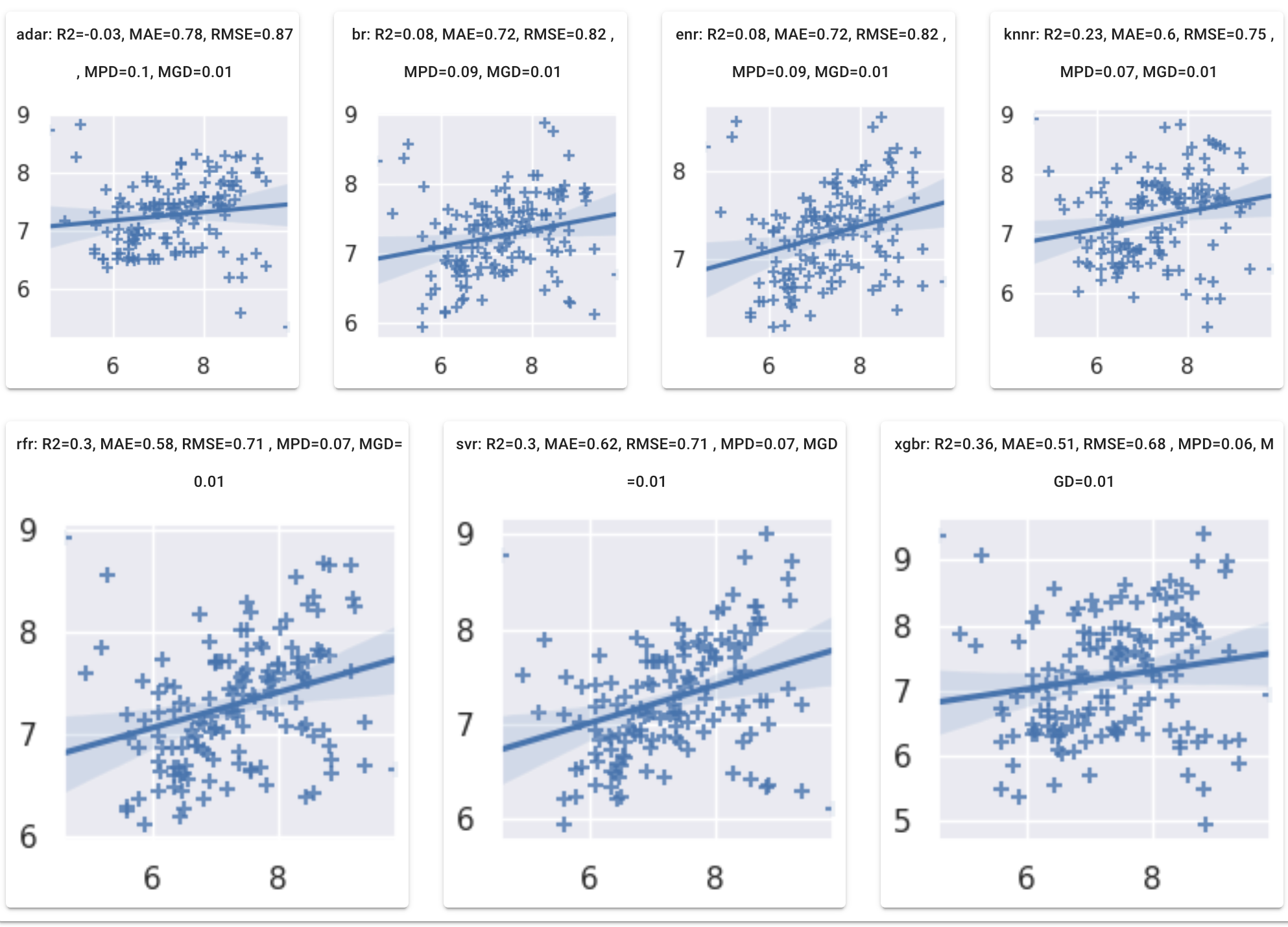


**A_3_AR – classification models**

For classification models, the dataset of 161 molecules was created with a threshold of 100 nM, which resulted in 83 active and 78 inactive molecules, that were used to build the models.


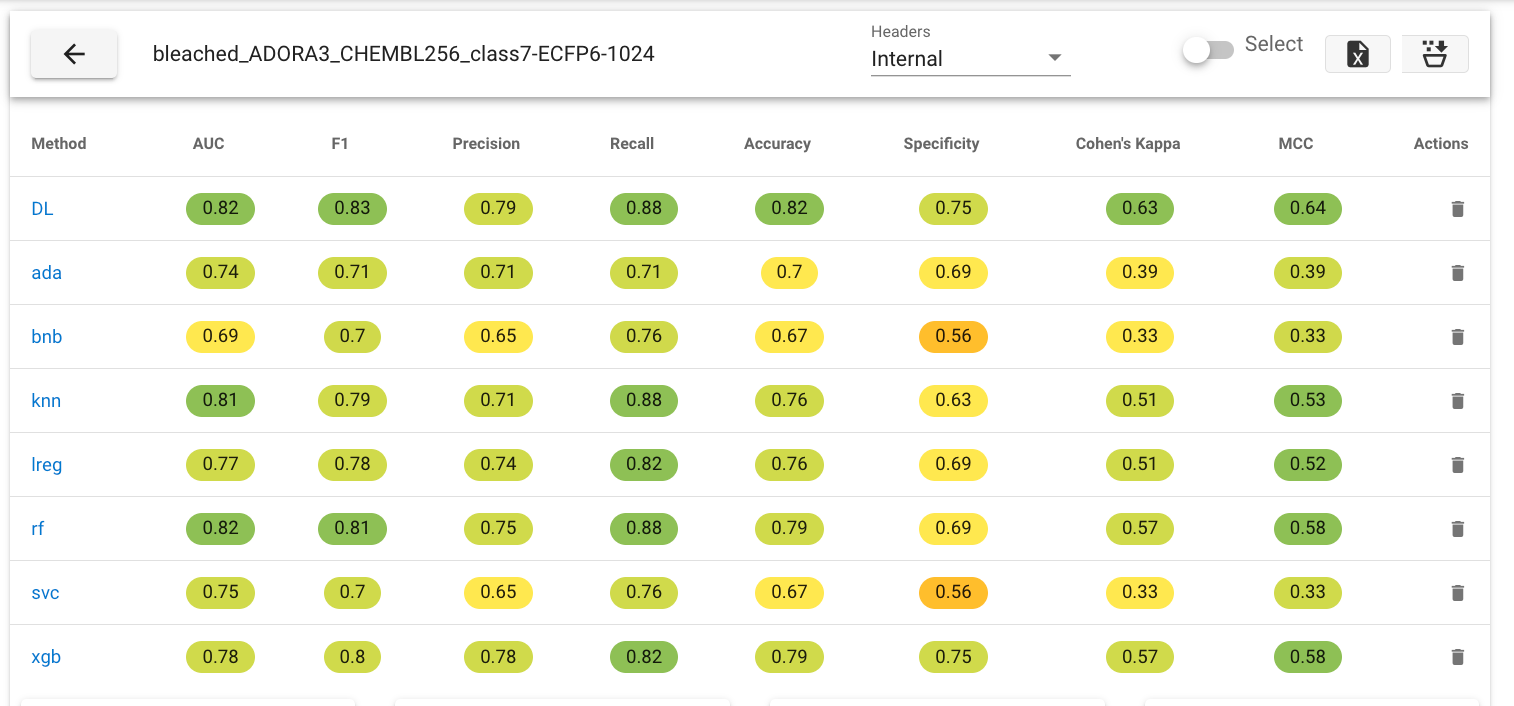


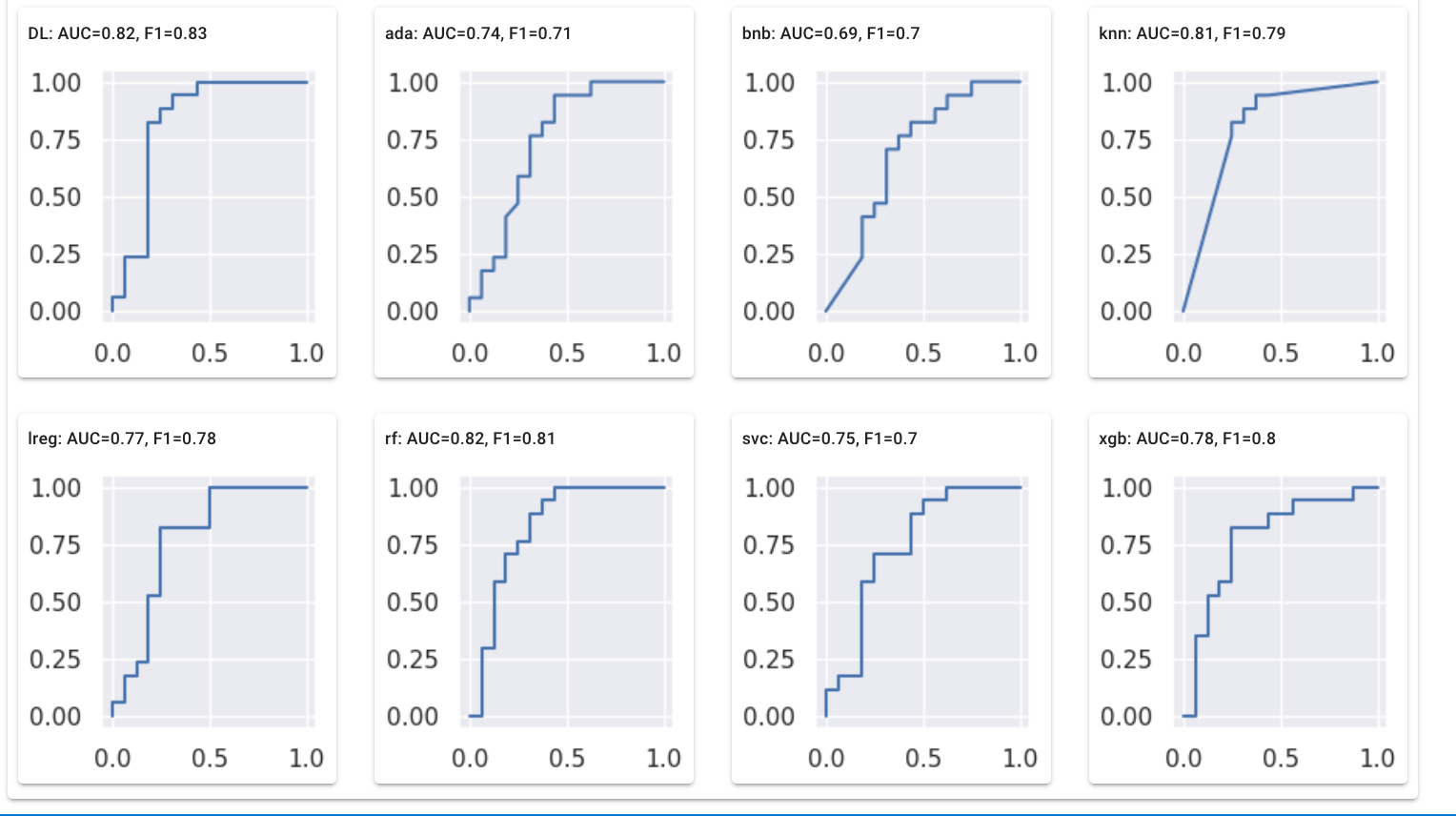


**Figure S5.** % Activation ADORA1 using ADORA1 - bla U2OS cells**. A)** Paroxetine and **B)** crisaborole.

**Figure S6.** Control dose response curve for the ADORA1 β-arrestin assay. **A)** Control dose response curve was performed for the ADORA1 Biosensor Assay. Data shown was normalized to the maximal and minimal response observed in the presence of control compound and vehicle respectively. **B)** Compound was tested in agonist mode with the ADORA1 Biosensor Assay. For agonist assay, data was normalized to the maximal and minimal response observed in the presence of control ligand and vehicle.

**Figure S7.** EC_50_ determination for crisaborole using cellular and nuclear receptor functional assays (Eurofins) for calcium influx assay. Data was normalized to the control. Control used in this assay: *N*^6^-cyclopentyladenosine (CPA), EC_50_ 91 nM.

**
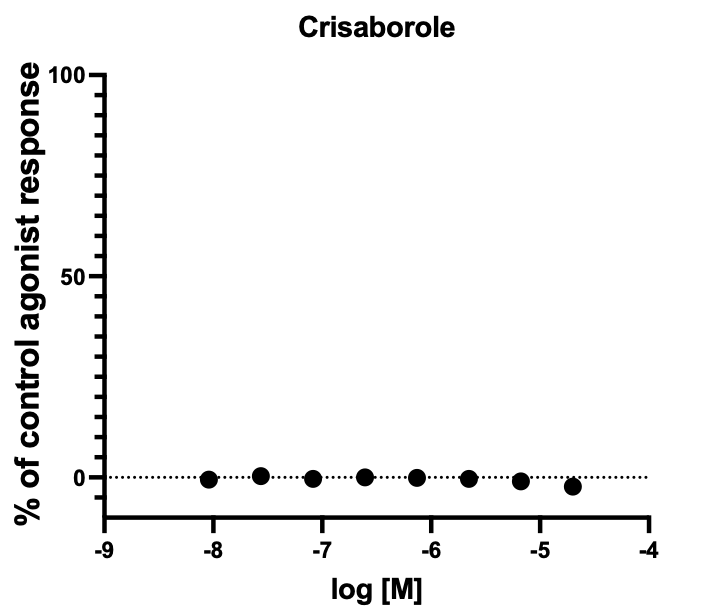
**

**Figure S8.** Representative A_1_AR modulators that have reached clinical trials to date. A. Trabodenoson. B. Neladenoson bialanate. C. T-62.(3, 46). A_2A_AR agonist Binodenoson(3). E) A_3_AR agonist piclidesonon(3).
